# Comparative Real-World Outcomes of OnabotulinumtoxinA and CGRP Monoclonal Antibodies in Chronic Migraine

**DOI:** 10.3390/jcm15134963

**Published:** 2026-06-25

**Authors:** Chun-Fu Lin, Chen-Chih Chung, Jia-Hung Chen, Nai-Fang Chi, Chaur-Jong Hu, Hung-En Huang, Chih-Chung Chen, Tu-Hsueh Yeh, James Cheng-Chung Wei, Hsun-Hua Lee

**Affiliations:** 1 Department of Neurology, Taipei Medical University Hospital, Taipei Medical University, Taipei 110301, Taiwan; 241009@h.tmu.edu.tw (C.-F.L.);; 2 Dizziness and Balance Disorder Center, Taipei Medical University Hospital, Taipei Medical University, New Taipei City 235061, Taiwan; 3 Department of Neurology, School of Medicine, College of Medicine, Taipei Medical University, Taipei 110301, Taiwan; 10670@s.tmu.edu.tw (C.-C.C.);; 4 Department of Neurology, Shuang Ho Hospital, Taipei Medical University, New Taipei City 235041, Taiwan; 5 Department of Neurology, Neurological Institute and Stroke Center, Taipei Veterans General Hospital, Taipei 112201, Taiwan; 6 School of Medicine, National Yang Ming Chiao Tung University, Taipei 112304, Taiwan; 7 Center for Health Data Science, Department of Medical Research, Chung Shan Medical University Hospital, Taichung 402306, Taiwan; 8 Institute of Medicine, Chung Shan Medical University, Taichung 402306, Taiwan; 9 Division of Allergy, Immunology & Rheumatology, Department of Internal Medicine, Chung Shan Medical University Hospital, Taichung 402306, Taiwan; 10 Graduate Institute of Integrated Medicine, China Medical University, Taichung 404328, Taiwan

**Keywords:** chronic migraine, onabotulinumtoxinA, CGRP monoclonal antibodies, real-world evidence, propensity score matching

## Abstract

**Background:** OnabotulinumtoxinA and calcitonin gene-related peptide (CGRP) monoclonal antibodies are widely used for chronic migraine prevention, but comparative real-world evidence on healthcare utilization remains limited. This study aimed to compare the association of onabotulinumtoxinA versus CGRP monoclonal antibodies with acute triptan prescription and migraine-related return visits in patients with chronic migraine. **Methods:** We conducted a retrospective cohort study using the TriNetX global federated electronic health record database from 2018 to 2024. Adults with chronic migraine who initiated onabotulinumtoxinA or a CGRP monoclonal antibody were matched 1:1 by propensity score. The primary outcomes were time to acute triptan prescription and time to first migraine-related return visit during follow-up. **Results:** After propensity score matching, 10,140 patients were included in each treatment group. OnabotulinumtoxinA was associated with a lower hazard of acute triptan prescription than CGRP monoclonal antibodies (hazard ratio 0.513, 95% confidence interval 0.481–0.546; *p* < 0.001), whereas migraine-related return visits were similar between groups (hazard ratio 1.008, 95% confidence interval 0.977–1.039; *p* = 0.69). **Conclusions:** In this multicenter real-world analysis, onabotulinumtoxinA was associated with a lower hazard of acute triptan prescription than CGRP monoclonal antibodies, while migraine-related return visits were comparable. These findings reflect treatment-related healthcare utilization patterns in routine practice and should be interpreted considering the limitations of retrospective electronic health record data.

## 1. Introduction

Chronic migraine is a disabling neurological disorder that imposes substantial individual, social, and healthcare burdens worldwide, with adverse effects on daily functioning, quality of life, work productivity, and healthcare utilization [1]. Preventive therapy is therefore a central component of long-term management, particularly in patients with frequent headache days, medication overuse, or repeated healthcare visits.

Among currently available preventive options, onabotulinumtoxinA and calcitonin gene-related peptide (CGRP) monoclonal antibodies are widely used for patients with chronic migraine who require more intensive preventive treatment [2,3]. Although both strategies are supported by real-world and clinical evidence [4,5,6], they differ in route of administration, treatment schedule, biological target, and potentially in the way treatment response is reflected in routine practice. OnabotulinumtoxinA is generally administered as clinician-delivered injections at regular intervals, whereas CGRP monoclonal antibodies are used as targeted mechanism-based therapies with different dosing schedules and drug-specific pharmacologic profiles [7,8,9,10].

Mechanistically, onabotulinumtoxinA is believed to reduce migraine activity by inhibiting the release of neurotransmitters and neuropeptides involved in peripheral sensitization and subsequent central pain processing, whereas CGRP monoclonal antibodies act by blocking the CGRP pathway, a key mediator in migraine pathophysiology [8,9,11,12]. Because these treatments act through distinct biological mechanisms and are delivered under different clinical circumstances, comparative evaluation in real-world settings is clinically relevant.

Previous observational studies have shown that anti-CGRP therapies and onabotulinumtoxinA can both improve migraine-related outcomes, including headache burden, treatment tolerability, and disability measures [4,5,6,7]. However, much of the existing literature has focused on symptom-centered outcomes, such as monthly migraine days, headache intensity, or patient-reported disability, while less attention has been paid to healthcare utilization outcomes that may better capture treatment patterns in routine care. In real-world databases, outcomes such as acute medication prescribing and return visits may serve as pragmatic indicators of disease control, treatment need, or follow-up intensity when detailed bedside clinical data are unavailable.

Direct head-to-head comparative evidence between onabotulinumtoxinA and CGRP monoclonal antibodies also remains limited, particularly in large multinational electronic health record datasets. In addition, important practical questions remain regarding whether one strategy is associated with lower acute medication use or different patterns of recurrent healthcare contact after treatment initiation. These questions are relevant to clinicians, patients, and health systems because treatment selection in chronic migraine often depends not only on efficacy, but also on comorbidity profile, access, reimbursement, follow-up structure, and anticipated healthcare utilization.

To address this gap, the present study used the TriNetX global federated electronic health record network to compare chronic migraine patients who initiated onabotulinumtoxinA with those who initiated a CGRP monoclonal antibody. After propensity score matching, the study evaluated two pragmatic time-to-event outcomes: acute triptan prescription and first migraine-related return visit during follow-up. Because TriNetX is based on routinely collected coded clinical data, the aim was not to assess detailed injection practice, headache-day frequency, or patient-reported symptom severity but rather to examine treatment-associated healthcare utilization patterns in a large real-world cohort [13,14].

## 2. Methods

### 2.1. Study Design and Data Collection

This retrospective cohort study was conducted using the TriNetX global research network, a federated platform that aggregates de-identified electronic health record data from multiple healthcare organizations. The study period extended from 1 January 2018 to 31 December 2024, and analyses were performed within the TriNetX analytics environment using the variables and functions available within the platform. The study was reported in accordance with the STROBE statement for observational studies.

The study was approved by the Taipei Medical University Joint Institutional Review Board (TMU-JIRB No. N202512042; approval date: 13 December 2025). The TriNetX query and analyses were conducted after IRB approval, and the data available for analysis were limited to records from 1 January 2018 to 31 December 2024. All data used in this study were de-identified before analysis, and the requirement for informed consent was waived because of the retrospective design and the use of de-identified records. TriNetX data are structured to comply with privacy requirements, and small cell counts are masked to reduce re-identification risk.

### 2.2. Study Population

Eligible patients were adults aged 18 years or older with chronic migraines identified by the corresponding ICD-10 diagnostic codes available in TriNetX (G43, G43.7, G43.70, and G43.709). Because TriNetX captures diagnoses through coded electronic health records (EHR)-based data, migraine subtypes such as aura status, status migrainosus, and intractability were identified only when explicitly coded and were otherwise not further subclassified. Patients were assigned to the onabotulinumtoxinA cohort or the CGRP monoclonal antibody cohort (erenumab, fremanezumab, galcanezumab, or eptinezumab) according to the first recorded treatment exposure during the study period. The index date was defined as the date of the first eligible treatment record. To reduce exposure overlap, patients who received both therapies within one month before or after the index date were excluded.

TriNetX is a federated platform that aggregates real-time electronic health records from multiple healthcare organizations. Missing values are retained in their original form without imputation or deletion [13]. In our analysis, follow-up was operationalized as the period from the index date to the patient’s last recorded clinical encounter in TriNetX, which served as the end of observable data availability [15]. All data were de-identified in accordance with Health Insurance Portability and Accountability Act (HIPAA) and the General Data Protection Regulation (GDPR) standards, and cell counts fewer than 10 were masked to protect patient privacy [16].

### 2.3. Exposure Definitions

Exposure was defined using treatment records in TriNetX. The onabotulinumtoxinA cohort was identified using the RxNorm code reported in the manuscript, and the CGRP monoclonal antibody cohort was defined using the corresponding RxNorm identifiers for the included anti-CGRP agents. Because TriNetX is an EHR-based platform, exposure ascertainment reflects recorded treatment documentation rather than protocolized trial administration. Detailed clinical information such as injection pattern, dose escalation, adherence, persistence, switching beyond the predefined window, and physician-specific delivery technique were not available for analysis.

### 2.4. Outcomes

The study evaluated two primary time-to-event outcomes during follow-up: time to acute triptan prescription and time to first migraine-related return visit. These endpoints were selected as pragmatic healthcare utilization proxies rather than direct symptom-based efficacy measures because patient-reported outcomes and detailed headache metrics are not available in TriNetX.

Time to acute triptan prescription was defined as the interval from the index date to the first post-index prescription record for any triptan included in the database query, including sumatriptan, zolmitriptan, rizatriptan, naratriptan, eletriptan, almotriptan, and frovatriptan. This outcome was interpreted as a proxy for breakthrough acute medication need rather than a direct measure of total triptan consumption, as TriNetX does not provide information on dose, days of supply, refill quantity, or cumulative use.

Migraine-related return visit was defined as the first post-index outpatient, emergency department, or inpatient encounter carrying a migraine-related ICD-10 diagnosis code (G43. *). Because some follow-up encounters may reflect planned monitoring rather than spontaneous symptom worsening, especially in patients treated with onabotulinumtoxinA at regular intervals, this endpoint was interpreted as treatment-related healthcare utilization rather than a pure indicator of disease recurrence.

### 2.5. Covariates and Matching

To reduce measured baseline imbalance between treatment groups, the analysis incorporated a broad set of covariates available in TriNetX. These included demographic factors such as age, sex, and race; lifestyle-related variables including nicotine dependence, tobacco use, and alcohol-related disorders; comorbidities such as hypertension, diabetes, sleep disorders, depression, and anxiety; prior healthcare utilization measures; concurrent medication use; and available laboratory values including C-reactive protein, hemoglobin, and estimated glomerular filtration rate.

Propensity score matching was performed in a 1:1 ratio using nearest-neighbor matching without replacement. Covariate balance after matching was assessed using standardized mean differences, with values below 0.1 considered indicative of acceptable balance. This matching strategy was intended to improve comparability between patients initiating onabotulinumtoxinA and those initiating a CGRP monoclonal antibody, while recognizing that residual confounding from unmeasured clinical factors may remain.

### 2.6. Statistical Analysis

Baseline characteristics were summarized before and after matching as appropriate for the TriNetX platform output. Time-to-event analyses were performed using Kaplan–Meier methods, with between-group comparisons assessed by log-rank testing. Cox proportional hazards regression was used to estimate hazard ratios and 95% confidence intervals for the outcomes of interest. A two-sided *p*-value below 0.05 was considered statistically significant.

Follow-up was defined from the index date until the last recorded clinical encounter in TriNetX, which represented the end of observable data availability for each patient. As a result, the study assessed associations within the captured EHR follow-up period rather than fixed protocol-driven follow-up intervals.

### 2.7. Sensitivity and Subgroup Analyses

To assess the robustness of the main findings, a sensitivity analysis broadened the diagnostic definition to include headache-related ICD-10 code R51. This approach was intended to test whether the primary findings were materially altered when a wider symptom-based coding framework was used. Prespecified subgroup analyses further explored associations across age, sex, and selected comorbidity strata. These subgroup analyses were considered exploratory and were interpreted cautiously given the limited clinical granularity inherent to coded EHR data.

### 2.8. Ethical Considerations

This study was approved by the Taipei Medical University Joint Institutional Review Board (TMU-JIRB No. N202512042). The requirement for informed consent was waived because of the retrospective design and use of fully de-identified data.

## 3. Results

### 3.1. Patient Cohort Characteristics

**Figure 1:**
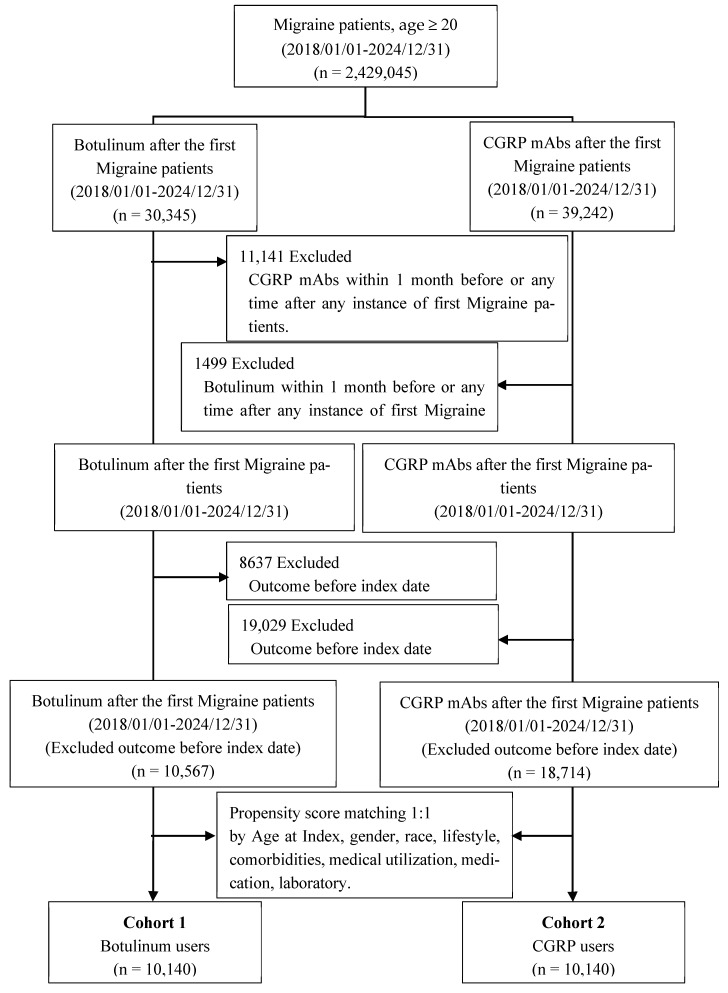
Flow diagram of cohort selection for patients with chronic migraine treated with onabotulinumtoxinA or CGRP monoclonal antibodies in the TriNetX database. Before matching: Botulinum users *n* = 10,567; CGRP mAbs users *n* = 18,714. After exclusions and 1:1 propensity score–matched cohorts, final cohorts *n* = 10,140 each. Arrows indicate cohort selection and exclusion flow. CGRP, calcitonin gene-related peptide; mAbs, monoclonal antibodies.

From 2,429,045 patients with migraine, 29,938 met chronic migraine and treatment eligibility criteria. After 1:1 propensity score matching, the final analytic cohorts included 10,140 patients in each group, with good baseline balance across measured covariates (all standardized mean differences < 0.1; Figure 1 and Table 1).

### 3.2. Primary Outcomes

**Figure 2:**
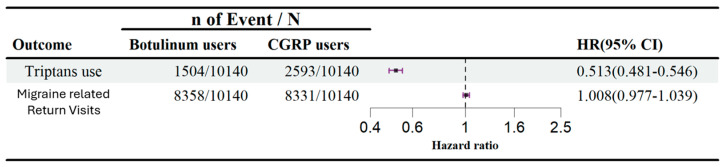
Comparative cumulative incidence of acute triptan prescriptions and migraine-related return visits over the one-year follow-up in patients treated with onabotulinumtoxinA versus CGRP monoclonal antibodies. Hazard ratios with 95% confidence intervals are shown. The dashed line indicates the reference value of HR = 1, and the symbols represent the hazard ratios with 95% confidence intervals for each outcome. CGRP, calcitonin gene-related peptide; HR, hazard ratio; CI, confidence interval.

**Figure 3:**
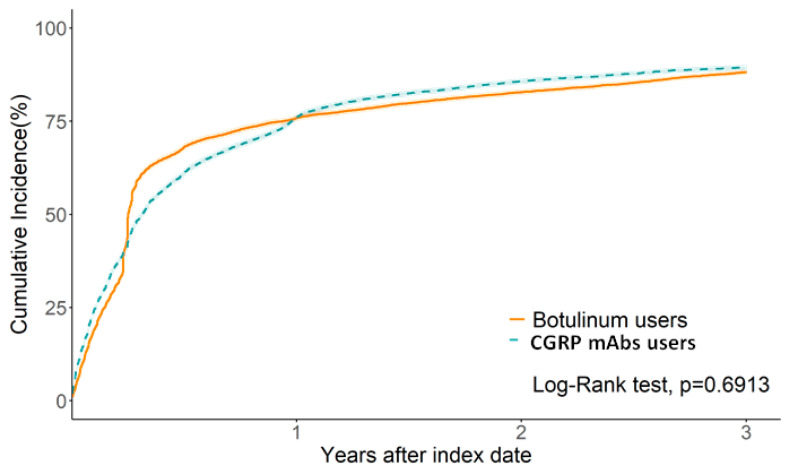
Kaplan–Meier cumulative risk curves for time to migraine-related return visit in patients with chronic migraine receiving onabotulinumtoxinA or CGRP monoclonal antibodies. CGRP, calcitonin gene-related peptide; mAbs, monoclonal antibodies.

OnabotulinumtoxinA was associated with a lower hazard of acute triptan prescription than CGRP monoclonal antibodies (HR 0.513, 95% CI 0.481–0.546, *p* < 0.001; Figure 2). Migraine-related return visits were similar between groups (HR 1.008, 95% CI 0.977–1.039, *p* = 0.69; Figure 3).

### 3.3. Sensitivity Analysis

**Figure 4:**
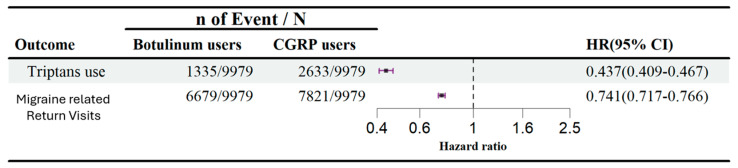
Incidence of acute triptan prescriptions and migraine-related return visits in the expanded cohort including patients with headache symptoms (ICD–10 R51), according to preventive treatment with onabotulinumtoxinA or CGRP monoclonal antibodies. The dashed line indicates the reference value of HR = 1, and the symbols represent the hazard ratios with 95% confidence intervals for each outcome. CGRP, calcitonin gene-related peptide; HR, hazard ratio; CI, confidence interval.

**Figure 5:**
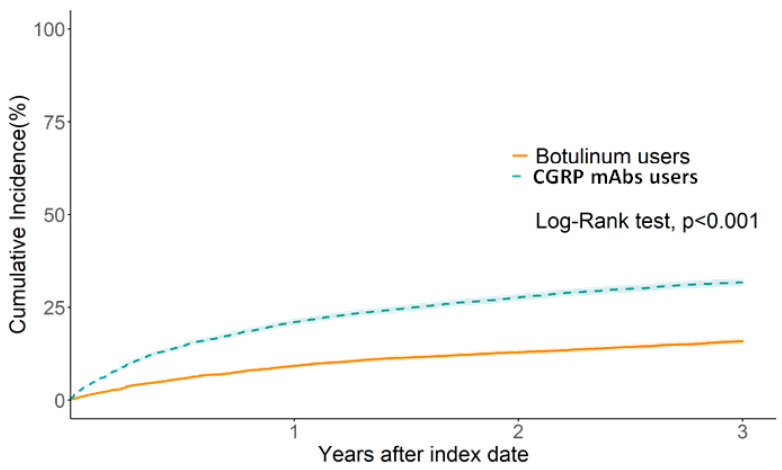
Kaplan–Meier cumulative risk curves for time to acute triptan prescription in the expanded headache cohort (ICD-10 R51). CGRP, calcitonin gene-related peptide; mAbs, monoclonal antibodies.

**Figure 6:**
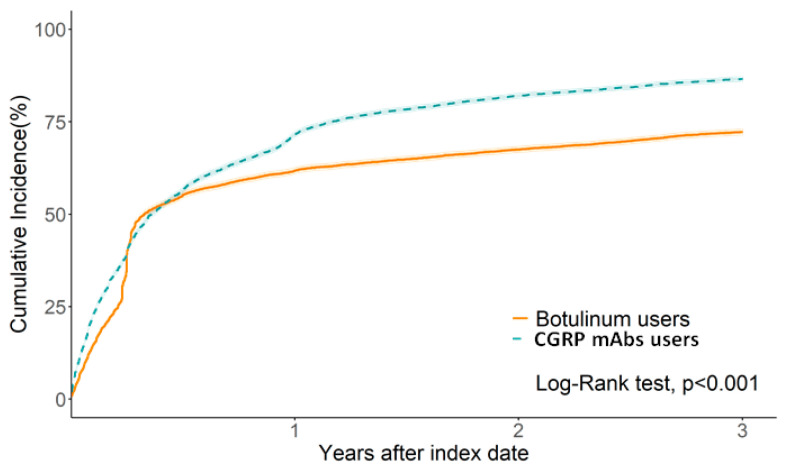
Kaplan–Meier cumulative risk curves for migraine-related return visits in the expanded headache cohort (ICD-10 R51). CGRP, calcitonin gene-related peptide; mAbs, monoclonal antibodies.

In the expanded cohort including R51.* headache codes, the association with acute triptan prescription remained consistent (HR 0.437, 95% CI 0.409–0.467). Findings for migraine-related return visits were also consistent with the primary analysis, although the expanded definition suggested a difference in healthcare utilization (Figure 4, Figure 5 and Figure 6).

### 3.4. Subgroup Analyses

**Figure 7:**
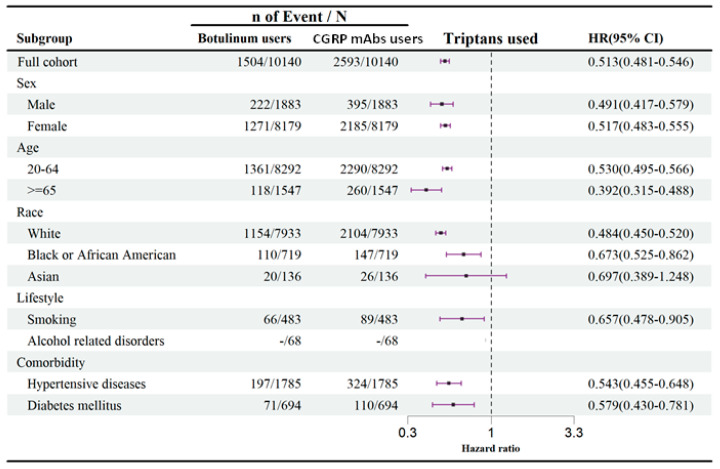
Subgroup analyses of the association between preventive treatment with onabotulinumtoxinA versus CGRP monoclonal antibodies and risk of acute triptan prescriptions. Forest plots display hazard ratios with 95% confidence intervals across strata of age, sex, race, smoking status, and major comorbidities. The dashed line indicates the reference value of HR = 1, and the symbols represent the hazard ratios with 95% confidence intervals for each outcome. CGRP, calcitonin gene-related peptide; mAbs, monoclonal antibodies; HR, hazard ratio; CI, confidence interval.

**Figure 8:**
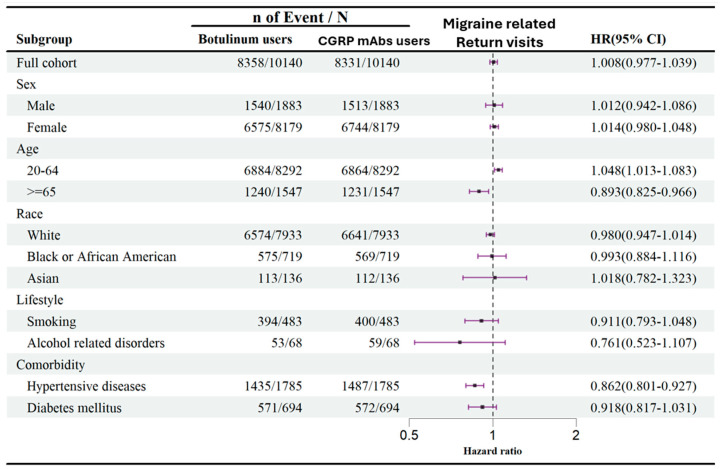
Subgroup analyses of the association between preventive treatment with onabotulinumtoxinA versus CGRP monoclonal antibodies and risk of migraine-related return visits. Forest plots display hazard ratios with 95% confidence intervals across strata of age, sex, race, smoking status, and major comorbidities. The dashed line indicates the reference value of HR = 1, and the symbols represent the hazard ratios with 95% confidence intervals for each outcome. CGRP, calcitonin gene-related peptide; mAbs, monoclonal antibodies; HR, hazard ratio; CI, confidence interval.

The lower hazard of acute triptan prescriptions with onabotulinumtoxinA was consistent across prespecified subgroups, including sex, age, race, smoking status, and major comorbidity strata (Figure 7). For migraine-related return visits, no overall difference was observed in the matched cohort, and subgroup patterns were heterogeneous but generally similar between treatments (Figure 8).

### 3.5. Summary of Findings

Across matched chronic migraine cohorts, onabotulinumtoxinA was associated with a lower hazard of acute triptan prescription than CGRP monoclonal antibodies, while migraine-related return visit rates were similar. These associations remained directionally consistent in sensitivity and subgroup analyses.

## 4. Discussion

### 4.1. Key Findings

In this multicenter real-world analysis of propensity score-matched patients with chronic migraine, initiation of onabotulinumtoxinA was associated with a lower hazard of acute triptan prescription compared with initiation of CGRP monoclonal antibodies, whereas the hazard of migraine-related return visit was similar between groups. These findings suggest that the two preventive strategies may differ in treatment-associated healthcare utilization patterns, even when broad measured baseline characteristics are balanced. These findings should be interpreted as associations rather than causal treatment effects, because the retrospective EHR-based design cannot fully account for unmeasured clinical factors, treatment selection, or care patterns [13,17].

### 4.2. Comparison with Previous Studies

Our findings add to prior real-world comparative studies of onabotulinumtoxinA and anti-CGRP therapies, including the RAMO study and related observational reports, which suggest that these preventive strategies may differ in clinically relevant ways despite both being effective in chronic migraine management [18,19,20,21]. However, much of the existing literature has focused on symptom-based outcomes, such as headache-day reduction, disability measures, or patient-reported benefit [19,20,22,23,24,25], including the RAMO study and the combination-therapy report by Blumenfeld et al., whereas the present study evaluated pragmatic healthcare utilization outcomes that are more readily captured in TriNetX, including acute triptan prescription and migraine-related return visits.

### 4.3. Interpretation of Utilization Outcomes

The lower hazard of acute triptan prescription observed in the onabotulinumtoxinA cohort may reflect lower subsequent acute medication need, but this interpretation remains indirect because TriNetX does not capture total medication consumption, refill behavior, or patient-reported symptom severity [13,17]. Accordingly, acute triptan prescription should be understood as a proxy for breakthrough acute medication use rather than a direct measure of clinical efficacy.

Migraine-related return visits also require cautious interpretation. In routine clinical practice, onabotulinumtoxinA is commonly administered at scheduled intervals, and some follow-up encounters may therefore reflect planned care rather than spontaneous symptom-driven utilization. For this reason, the return-visit outcome in this study is better interpreted as treatment-linked healthcare utilization than as a pure marker of disease activity.

### 4.4. Subgroup Interpretation

Although subgroup analyses suggested possible heterogeneity by age and comorbidity status, these findings should be considered exploratory, as the current dataset does not provide sufficient clinical granularity to determine whether the patterns reflect true treatment-response differences, underlying disease severity, treatment selection, or follow-up practices.

### 4.5. Strengths and Limitations

The strengths of this multicenter TriNetX analysis (*n* = 20,280) include a large propensity score-matched cohort drawn from diverse US healthcare systems and the use of pragmatic endpoints that complement traditional headache-day metrics from clinical trials by reflecting real-world healthcare utilization.

Despite these strengths, several limitations should be acknowledged. First, because TriNetX is based on de-identified EHR data, detailed injection information, including injection sites and dose, was unavailable, and the database also does not capture clinician technique or physician experience, both of which may influence treatment delivery and outcomes. Second, patient-reported outcomes such as pain severity, headache-day burden, and validated disability measures were not available, and the database does not allow precise assessment of total medication consumption, refill behavior, or days of supply. Third, migraine-related return visits may partly reflect scheduled follow-up care, particularly in the onabotulinumtoxinA group, which may affect time-to-event interpretation. Fourth, grouping different CGRP monoclonal antibodies together may have obscured drug-specific differences. Finally, as with any retrospective EHR study, residual confounding, coding misclassification, and incomplete capture of care delivered outside participating healthcare systems remain possible.

## 5. Conclusions

This multicenter TriNetX analysis of propensity score-matched patients found that onabotulinumtoxinA was associated with a lower hazard of acute triptan prescriptions than CGRP monoclonal antibodies in chronic migraine, while migraine-related return visit rates were comparable. These findings should be interpreted as observational associations reflecting healthcare utilization patterns rather than direct measures of treatment efficacy. Further prospective studies are needed to confirm these observations.

### Clinical Implications

Real-world associations suggest comorbidity- and age-stratified treatment consideration. Clinicians should weigh acute medication control versus healthcare utilization patterns alongside individual patient characteristics and local reimbursement criteria when selecting preventive therapies for chronic migraine.

## Figures and Tables

**Table 1:** Baseline characteristics of patients with chronic migraine treated with onabotulinumtoxinA versus CGRP monoclonal antibodies before and after propensity score matching. Data are presented as mean ± standard deviation or number (percentage), as appropriate. Std diff indicates standardized difference between groups; values less than 0.1 denote adequate balance after matching. PPHH–SPSC, persons with potential health hazards related to socioeconomic and psychosocial circumstances; CKD–EPI, Chronic Kidney Disease Epidemiology Collaboration; CGRP, calcitonin gene-related peptide; mAbs, monoclonal antibodies. All variables were included in the propensity score model using 1:1 nearest-neighbor matching without replacement.

	Before Matching			After Matching		
	Botulinum Users (*n* = 10,567)	CGRP mAbs Users (*n* = 18,714)	Std Diff	Botulinum Users (*n* = 10,140)	CGRP mAbs Users (*n* = 10,140)	Std Diff
**Age at index**						
Mean ± SD	50.1 ± 14.8	45.9 ± 14.4	0.2859	49.5 ± 14.7	49.3 ± 14.5	0.0154
**Gender, *n* (%)**						
Female	8511 (80.5%)	15,328 (81.9%)	0.0349	8191 (80.8%)	8238 (81.2%)	0.0118
Male	2046 (19.4%)	3373 (18.0%)	0.0343	1941 (19.1%)	1894 (18.7%)	0.0118
Unknown Gender	10 (0.1%)	13 (0.1%)	0.0088	10 (0.1%)	10 (0.1%)	0.0000
**Race, *n* (%)**						
White	8144 (77.1%)	14,581 (77.9%)	0.0202	7836 (77.3%)	7849 (77.4%)	0.0031
Black or African American	755 (7.1%)	1537 (8.2%)	0.0401	737 (7.3%)	771 (7.6%)	0.0128
Asian	180 (1.7%)	263 (1.4%)	0.0241	168 (1.7%)	160 (1.6%)	0.0063
Other Race	417 (3.9%)	830 (4.4%)	0.0244	409 (4.0%)	421 (4.2%)	0.0060
Unknown Race	978 (9.3%)	1357 (7.3%)	0.0729	901 (8.9%)	857 (8.5%)	0.0154
**Medical utilization, *n* (%)**						
Office or Other Outpatient Services	4769 (45.1%)	5530 (29.6%)	0.3264	4373 (43.1%)	4489 (44.3%)	0.0231
Emergency Department Services	1164 (11.0%)	1908 (10.2%)	0.0266	1118 (11.0%)	1135 (11.2%)	0.0053
Preventive Medicine Services	634 (6.0%)	820 (4.4%)	0.0730	594 (5.9%)	591 (5.8%)	0.0013
Hospital Inpatient and Observation Care Services	503 (4.8%)	651 (3.5%)	0.0645	461 (4.5%)	465 (4.6%)	0.0019
**Lifestyles** **, *n* (%)**						
Nicotine dependence	496 (4.7%)	787 (4.2%)	0.0237	475 (4.7%)	502 (5.0%)	0.0124
Tobacco use	132 (1.2%)	173 (0.9%)	0.0313	119 (1.2%)	128 (1.3%)	0.0081
Alcohol related disorders	102 (1.0%)	137 (0.7%)	0.0254	94 (0.9%)	97 (1.0%)	0.0031
**Social economic, *n* (%)**						
PPHH-SPSC	92 (0.9%)	137 (0.7%)	0.0155	87 (0.9%)	92 (0.9%)	0.0053
**Comorbidities, *n* (%)**						
Hypertensive diseases	2017 (19.1%)	2466 (13.2%)	0.1612	1820 (17.9%)	1844 (18.2%)	0.0062
Other anxiety disorders	1764 (16.7%)	2263 (12.1%)	0.1314	1616 (15.9%)	1616 (15.9%)	0.0000
Disorders of lipoprotein metabolism and other lipidemias	1593 (15.1%)	1921 (10.3%)	0.1450	1430 (14.1%)	1445 (14.3%)	0.0042
Sleep disorders	1527 (14.5%)	1777 (9.5%)	0.1531	1371 (13.5%)	1375 (13.6%)	0.0012
Depressive episode	1377 (13.0%)	1577 (8.4%)	0.1492	1233 (12.2%)	1198 (11.8%)	0.0106
Overweight and obesity	867 (8.2%)	1403 (7.5%)	0.0263	828 (8.2%)	864 (8.5%)	0.0128
Diabetes mellitus	747 (7.1%)	1164 (6.2%)	0.0341	723 (7.1%)	729 (7.2%)	0.0023
Asthma	752 (7.1%)	964 (5.2%)	0.0820	682 (6.7%)	698 (6.9%)	0.0063
Fibromyalgia	621 (5.9%)	631 (3.4%)	0.1195	541 (5.3%)	515 (5.1%)	0.0115
Ischemic heart diseases	532 (5.0%)	624 (3.3%)	0.0850	481 (4.7%)	489 (4.8%)	0.0037
Diseases of arteries, arterioles and capillaries	460 (4.4%)	506 (2.7%)	0.0895	408 (4.0%)	423 (4.2%)	0.0075
Cerebral infarction	314 (3.0%)	329 (1.8%)	0.0799	261 (2.6%)	266 (2.6%)	0.0031
Heart failure	206 (1.9%)	243 (1.3%)	0.0515	179 (1.8%)	199 (2.0%)	0.0146
Transient cerebral ischemic attacks and related syndromes	105 (1.0%)	172 (0.9%)	0.0077	102 (1.0%)	105 (1.0%)	0.0029
**Medication/procedure, *n* (%)**						
Psychoanaleptics	4369 (41.3%)	6125 (32.7%)	0.1791	4086 (40.3%)	4191 (41.3%)	0.0211
Psycholeptics	3594 (34.0%)	4582 (24.5%)	0.2106	3301 (32.6%)	3328 (32.8%)	0.0057
Opioids	2505 (23.7%)	3013 (16.1%)	0.1914	2265 (22.3%)	2270 (22.4%)	0.0012
Glucocorticoids	2184 (20.7%)	2634 (14.1%)	0.1747	1973 (19.5%)	1958 (19.3%)	0.0037
**Laboratory**						
Hemoglobin [Mass/volume] in Blood						
	13.2 ± 1.67	13.4 ± 1.61	0.0743	13.2 ± 1.67	13.3 ± 1.6	0.0559
Glomerular filtration rate/1.73 sq M.predicted [Volume Rate/Area] in Serum, Plasma or Blood by Creatinine-based formula (CKD-EPI)						
	85.8 ± 23	89 ± 22.8	0.1394	86.6 ± 22.9	86.5 ± 22.5	0.0069
Erythrocyte sedimentation rate						
	18 ± 19	17.5 ± 17.9	0.0274	18.3 ± 19.5	17.6 ± 17.7	0.0357
C reactive protein [Mass/volume] in Serum, Plasma or Blood						
	13.1 ± 35.2	11.1 ± 31	0.0595	13.4 ± 35.9	11.8 ± 34.3	0.0453

## Data Availability

The data that support the findings of this study are available from TriNetX, but restrictions apply to the availability of these data, which were used under license for the current study and are therefore not publicly available. Data may be available from the authors upon reasonable request and with permission from TriNetX.

## References

[1] (2024). GBD 2021 Nervous System Disorders Collaborators. Global, regional, and national burden of disorders affecting the nervous system, 1990–2021: A systematic analysis for the Global Burden of Disease Study 2021. Lancet Neurol..

[2] Kung D., Rodriguez G., Evans R. (2023). Chronic migraine diagnosis and management. Neurol. Clin..

[3] Lu J., Zhang Q., Guo X., Liu W., Xu C., Hu X., Ni J., Lu H., Zhao H. (2021). Calcitonin gene-related peptide monoclonal antibody versus Botulinum Toxin for the preventive treatment of chronic migraine: Evidence from indirect treatment comparison. Front. Pharmacol..

[4] Ashina M., Mitsikostas D.D., Amin F.M., Kokturk P., Schankin C.J., Sahin G., Pozo-Rosich P., Dorman P.J., Nedal T., Poole A.C. (2023). Real-world effectiveness of fremanezumab for the preventive treatment of migraine: Interim analysis of the pan-European, prospective, observational, phase 4 PEARL study. Cephalalgia.

[5] Montisano D.A., Giossi R., Canella M., Altamura C., Marcosano M., Vernieri F., Raggi A., Grazzi L. (2024). Reducing the impact of headache and allodynia score in chronic migraine: An exploratory analysis from the real-world effectiveness of anti-CGRP monoclonal antibodies compared to Onabotulinum Toxin A (RAMO) study. Toxins.

[6] Alabdali M.M., Rafique N., AlDossary D.A., Alalloush R.S., AlHemli H.A., Zeerak M., Latif R., Ibrahim Al-Asoom L., Abdulrahman AlSunni A., Mohammed Salem A. (2024). Direct comparison of treatment outcome between the onabotulinumtoxinA and calcitonin gene-related peptide monoclonal antibody in migraine patients. J. Clin. Med. Res..

[7] Pellesi L. (2023). Combining onabotulinumtoxin A with a CGRP antagonist for chronic migraine prophylaxis: Where do we stand?. Front. Pain Res..

[8] Corasaniti M.T., Lawrence G.W., Bagetta G., Iannacchero R., Tarsitano A., Monteleone A., Pagliaro M., Tonin P., Sandrini G., Nicotera P. (2023). Combination of anti-CGRP/CGRP-R mAbs with onabotulinumtoxin A as a novel therapeutic approach for refractory chronic migraine: A retrospective study of real-world clinical evidence and a protocol for a double-blind, randomized clinical trial to establish the efficacy and safety. Front. Pharmacol..

[9] Salim A., Hennessy E., Sonneborn C., Hogue O., Biswas S., Mays M., Suneja A., Ahmed Z., Mata I.F. (2024). Synergism of anti-CGRP monoclonal antibodies and onabotulinumtoxinA in the treatment of chronic migraine: A real-world retrospective chart review. CNS Drugs.

[10] Wu J.W., Yang C.P., Treatment Guideline Subcommittee of the Taiwan Headache Society (2022). 2022 Taiwan guidelines for preventive treatment of migraine. Acta Neurol. Taiwan.

[11] Aguilar-Shea A.L., Membrilla M.D.J., Diaz-de-Teran J. (2022). Migraine review for general practice. Aten. Primaria.

[12] Tzankova V., Becker W.J., Chan T.L.H. (2023). Pharmacologic prevention of migraine. CMAJ.

[13] Ludwig R.J., Anson M., Zirpel H., Thaci D., Olbrich H., Bieber K., Kridin K., Dempfle A., Curman P., Zhao S.S. (2025). A comprehensive review of methodologies and application to use the real-world data and analytics platform TriNetX. Front. Pharmacol..

[14] Lin T.L., Fan Y.H., Fan K.S., Juan C.K., Chen Y.J., Wu C.Y. (2024). Reduced atopic march risk in pediatric atopic dermatitis patients prescribed dupilumab versus conventional immunomodulatory therapy: A population-based cohort study. J. Am. Acad. Dermatol..

[15] Schneeweiss S., Patorno E. (2021). Conducting real-world evidence studies on the clinical outcomes of diabetes treatments. Endocr. Rev..

[16] TriNetX Privacy Policy. https://trinetx.com/trust-center/privacy/privacy-policy/.

[17] Nassar M., Abosheaishaa H., Elfert K., Beran A., Ismail A., Mohamed M., Misra A., Essibayi M.A., Altschul D.J., Azzam A.Y. (2025). TriNetX and real-world evidence: A critical review of its strengths, limitations, and bias considerations in clinical research. ASIDE Intern. Med..

[18] Grazzi L., Giossi R., Montisano D.A., Canella M., Marcosano M., Altamura C., Vernieri F. (2024). Real-world effectiveness of anti-CGRP monoclonal antibodies compared to onabotulinumtoxinA (RAMO) in chronic migraine: A retrospective, observational, multicenter, cohort study. J. Headache Pain.

[19] Ray J.C., Dalic L., Baker J., Cheng S., Hutton E.J., Matharu M. (2024). Twelve-month efficacy of CGRP monoclonal antibodies and predictive value of short-term response: Results of an Australian multicentre study. BMJ Neurol. Open.

[20] Wang Y.-F., Yang F.-C., Chen L.-A., Chang T.-Y., Su H.-C., Yang C.-P., Tu Y.-H., Tzeng Y.-S., Chen S.-P., Fuh J.-L. (2024). Comparative effectiveness and tolerability of calcitonin gene-related peptide (CGRP) monoclonal antibodies and onabotulinumtoxinA in chronic migraine: A multicenter, real-world study in Taiwan. Eur. J. Neurol..

[21] Salim A., Biswas S., Sonneborn C., Hogue O., Hennessy E., Mays M., Suneja A., Ahmed Z., Mata I.F. (2025). Efficacy and tolerability of anti-CGRP monoclonal antibodies in patients aged ≥65 years with daily or nondaily migraine. Neurol. Clin. Pract..

[22] Goenka A., Grace Yu S., Chikkannaiah M., George M.C., MacDonald S., Stolfi A., Kumar G. (2022). Generalized anxiety disorder: A predictor for poor responsiveness to botulinum toxin type A therapy for pediatric migraine. Pediatr. Neurol..

[23] Horvat D.E., Shields J.M., Young W.W.C., Eye P.G. (2023). Botulinum toxin for pediatric migraine: A retrospective multisite cohort study. Pediatr. Neurol..

[24] Gómez-Dabó L., Caronna E., Mas-de-Les-Valls R., Gallardo V.J., Alpuente A., Torres-Ferrus M., Pozo-Rosich P. (2024). Effectiveness and safety of onabotulinumtoxinA in adolescent patients with chronic migraine. Toxins.

[25] Blumenfeld A.M., Frishberg B.M., Schim J.D., Iannone A., Schneider G., Yedigarova L., Manack Adams A. (2021). Real-world evidence for control of chronic migraine patients receiving CGRP monoclonal antibody therapy added to onabotulinumtoxinA: A retrospective chart review. Pain Ther..

